# Supplementary material for: China-Pakistan economic corridor and its impact on rural development and human life sustainability. Observations from rural women
Source: PLoS One. 2020 Oct 2;15(10):e0239546. doi: 10.1371/journal.pone.0239546 (PMC7531834; doi:10.1371/journal.pone.0239546)

Appendix

Please circle the relevant option given below:

My age:

1. 20 to 30 years
2. 31 to 40 years
3. 41 to 50 years
4. 51 to 60 years
5. 61 and above

My education:

1. Matriculation
2. Secondary school
3. Bachelor
4. Master
5. MS MPhil
6. PhD

Please circle relevant option against each statement on the basis of strongly disagree1, disagree2, neutral3, agree4 and strongly agree5.

| **CPEC development** |
| --- |
| 1. Supporting services development (travel agency, hotel, restaurants and entertainment) |
| 1. Development of CPEC is vital to the area |
| 1. CPEC would play an important role in the area economy |
| 1. CPEC attract more people to the area |
| 1. CPEC should develop soon to improve the overall infrastructure |
| **Quality of Life** |
| 1. The quality of my life would generally enhance due to CPEC development |
| 1. This community would become a desirable place to live due to CPEC development |
| 1. The opportunities for leisure or recreation activities in this community would increase due to CPEC development |
| 1. The regional economy would boost due to CPEC development. |
| **Employment Opportunities** |
| 1. CPEC would generate employment opportunities in the area |
| 1. CPEC would create chances for a person to find a good job. |
| 1. CPEC would generate new business opportunities in the area |
| 1. Employment wages would become better in CPEC jobs |
| 1. CPEC would provide employment and skills for improved livelihood opportunities. |
| 1. More employment opportunities mean less crime. |
| **Rural Development** |
| 1. CPEC would improve electricity in the area and would reduce the loading shedding |
| 1. CPEC would improve the process of mobile communication and telecommunication process |
| 1. CPEC would build new schools and educational institutions in the area |
| 1. CPEC would progress food industry and quality food in the region |
| 1. CPEC would build new hospital and would improve health facilities in the area |
| 1. CPEC would benefit agriculture land and agriculture market in the area |
| **Self-Enhancement** |
| 1. Social power (control over others) |
| 1. Dominance (able to defeat others) |
| 1. Wealth (material possessions, money) |
| 1. Authority (the right to lead or command) |
| 1. Influence (having an impact on people and events) |


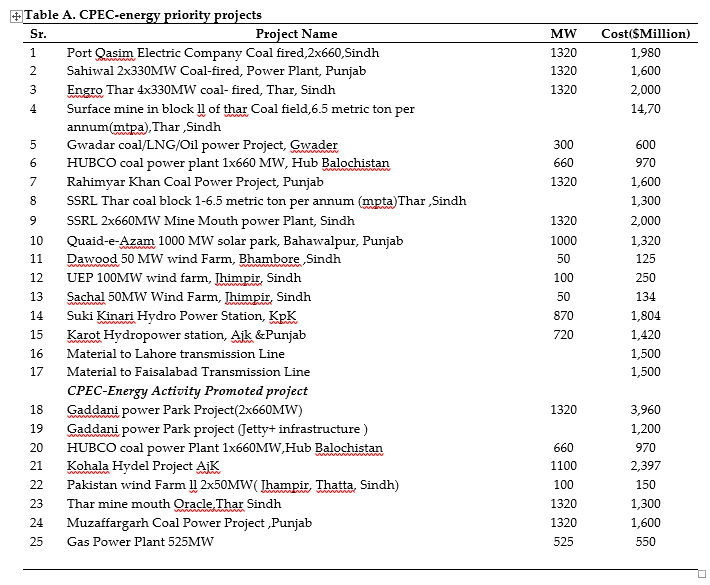


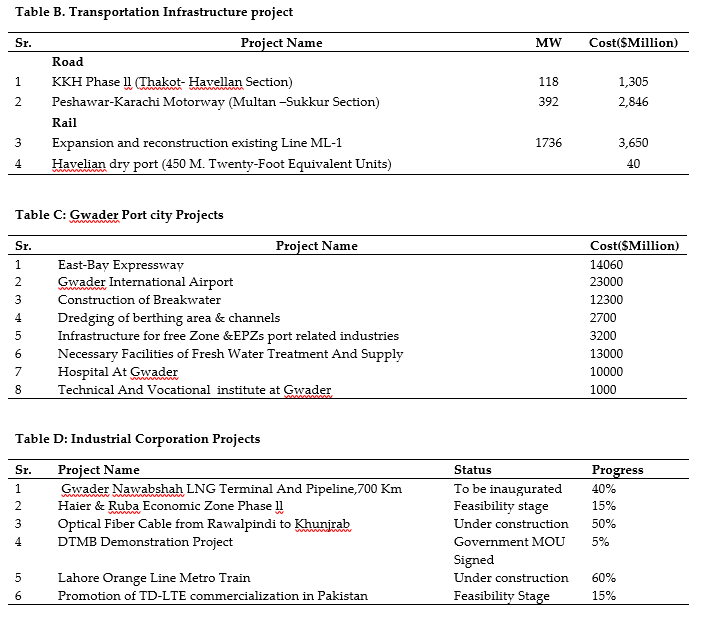


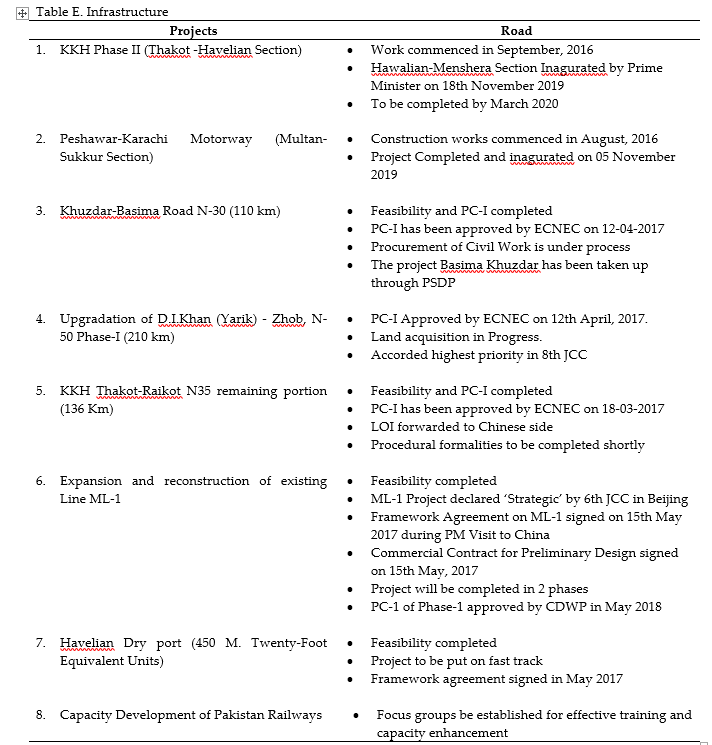

Supplement: S1 Appendix — (DOCX) [file pone.0239546.s001.docx]
