# Supplementary material for: China-Pakistan economic corridor and its impact on rural development and human life sustainability. Observations from rural women
Source: PLoS One. 2020 Oct 2;15(10):e0239546. doi: 10.1371/journal.pone.0239546 (PMC7531834; doi:10.1371/journal.pone.0239546)
Supplement: Attachment — Submitted filename: PONE-D-20-12198.docx [file pone.0239546.s002.docx]

This paper studied the impacts of CPEC on rural development and human life sustainability. It is a useful and important study, which is well supported by the questionairs and well referenced. However, there are quite many questions about the study.

(1) The structure is quite confusing. For example, *Section 4.2.1* comes after *Section 3.3*, and the *Section 4.1 is* after *Section 4.2.13* ?
(2) It would be better that declare the theoretical framework part together, which may involve *Section 2* and *Section 4* in the manuscript.
(3) *Section 6* is not really results, but description of data.
(4) I could not find the analysis part, but go strait from data description to conclusion.
(5) Why use so many semicolon punctuation in the manuscript? For example, the semicolon punctuation in the title.
(6) As you mentioned, there were 302 responses. However, in *Section 6.5,* most questions only get response from 19-32 respondents. Why?
