# Supplementary material for: China-Pakistan economic corridor and its impact on rural development and human life sustainability. Observations from rural women
Source: PLoS One. 2020 Oct 2;15(10):e0239546. doi: 10.1371/journal.pone.0239546 (PMC7531834; doi:10.1371/journal.pone.0239546)
Supplement: Attachment — Submitted filename: Response to the reviewers comments.docx [file pone.0239546.s004.docx]

Thank you once again the Editor and Reviewers for their time and comments since submission to finalization. We have incorporated all the comments in the revised manuscript. We hope the latest version is ready for publication.

Grammatical and typing Mistake: We have proofread the paper again and have detected the grammatical and typing mistakes in the manuscript. We believe that the revised version is free of errors.

6. Review Comments to the Author

Reviewer #1: All of the concerns have been addressed. Just a few suggestions to reduce ambiguity and to improve the quality of this manuscript.

The sampling method needs to be mentioned in the methodology section. For instance, only educated women have been selected and approached for questionnaire needs to be mentioned like it is mentioned that other 32 women who never went to a school were interviewed. This will improve the understanding of the reader that how the sampling was conducted.

Ans: Thank you for the useful comment. CPEC is initiated recently, it is a new project and is not a general project like others. Therefore, is argue that in general, an illiterate person may not able to understand and predict the benefits of the CPEC. During the survey and interview, we also acknowledge that most of the illiterate women do not know the CPEC. So it was difficult to receive relevant information for our research. Hence, we only interview those illiterate women who have some sort of knowledge of CPEC.

“For instance, educated people have more knowledge and understanding of CPEC and other mega projects and can predict future benefits. However, to articulate the results in a better way, we also gathered information from 32 illiterate women. We interviewed only those illiterate women in rural areas who were aware of the CPEC. Since it was difficult to know the exact number of educated women in the areas. Therefore, we used a convenient sampling approach to complete the data collection. The convenient sampling method was also appropriate due to the wide geographic distance in these areas. Hence, both educated (for questionnaire) and illiterate (for interview) women were approached conveniently. Moreover, the respondents were informed in starting of the survey that “the survey is a volunteer and your information can help us in completing the research”.

Reviewer #2: The structure is still very confusing. For example, Section 4.2.1 comes after Section 3.3, and the Section 4.1 is after Section 4.2.13, Section 1 is after Section 5?

Ans: Thank you once again for pointing out the mistakes, perhaps happened due to typing mistakes. We have corrected the numbering in the revised manuscript.

Thank you.
