# Supplementary material for: China-Pakistan economic corridor and its impact on rural development and human life sustainability. Observations from rural women
Source: PLoS One. 2020 Oct 2;15(10):e0239546. doi: 10.1371/journal.pone.0239546 (PMC7531834; doi:10.1371/journal.pone.0239546)
Supplement: Attachment — Submitted filename: Response to the reviewers comments.docx [file pone.0239546.s005.docx]

Thank you once again the Editor and Reviewers for their time and comments

We have received only one comment on the format of the manuscript. We have carefully reviewed the format and have merged some misleading numbering and headings. We believe that the revised format is free of vague and overlapping.

We hope that the Editor will approve the paper and will provide his feedback soon. We are waiting to hear the good news soon, as to submit the paper for an award in our university.

Thank you once again for your time and consideration.
